# Variation in Facility-Level Rates of All-Cause and Potentially Preventable 30-Day Hospital Readmissions Among Medicare Fee-for-Service Beneficiaries After Discharge From Postacute Inpatient Rehabilitation

**DOI:** 10.1001/jamanetworkopen.2019.17559

**Published:** 2019-12-13

**Authors:** Matt P. Malcolm, Addie Middleton, Allen Haas, Kenneth J. Ottenbacher, James E. Graham

**Affiliations:** 1 Department of Occupational Therapy, Colorado State University, Fort Collins; 2 Colorado School of Public Health, Aurora; 3 Division of Physical Therapy, Medical University of South Carolina, Charleston; 4 University of Texas Medical Branch, Galveston

## Abstract

**Importance:**

The Improving Medicare Post-Acute Care Transformation Act of 2014 mandated a quality measure of potentially preventable 30-day hospital readmission for inpatient rehabilitation facilities (IRFs). Examining IRF performance nationally may help inform health care quality initiatives for Medicare beneficiaries.

**Objective:**

To examine variation in Centers for Medicare & Medicaid Services Quality Reporting Program measures for US facility-level risk-adjusted all-cause and potentially preventable hospital readmission rates after inpatient rehabilitation.

**Design, Setting, and Participants:**

This cohort study of Medicare claims data included 454 378 Medicare beneficiaries discharged from 1162 IRFs between June 1, 2013, and July 1, 2015. Data were analyzed March 23, 2018, through June 24, 2019.

**Main Outcomes and Measures:**

All-Cause Unplanned Readmission Measure for 30 Days Post Discharge From Inpatient Rehabilitation Facilities and the Potentially Preventable 30-Day Post-Discharge Readmission Measure for Inpatient Rehabilitation. Specifications from the Centers for Medicare & Medicaid Services were followed to identify the cohort, define outcomes, and calculate risk-standardized facility-level rates.

**Results:**

Among a cohort of 454 378 patients, the mean (SD) age was 76.2 (10.6) years and 263 546 (58.0%) were women. The all-cause readmission rate was 12.3% (95% CI, 12.2%-12.4%), and the potentially preventable readmission rate was 5.3% (95% CI, 5.3%-5.4%). Across 1162 included IRFs, risk-standardized all-cause readmission rates ranged from 10.1% (95% CI, 8.9%-11.6%) to 15.9% (95% CI, 13.6-18.6%) and potentially preventable readmission rates ranged from 4.3% (95% CI, 3.7%-5.4%) to 7.3% (95% CI, 5.7%-8.3%). Using the All-Cause Unplanned Readmission Measure for 30 Days Post Discharge From Inpatient Rehabilitation Facilities, 16 IRFs (1.4%) had 95% CIs above the national mean rate, 1137 IRFs (97.9%) had 95% CIs containing the national mean rate, and 9 IRFs (0.8%) had 95% CIs below the national mean rate. Using the Potentially Preventable 30-Day Post-Discharge Readmission Measure for Inpatient Rehabilitation, 8 IRFs (0.7%) had 95% CIs above the national mean rate, 1153 IRFs (99.2%) had 95% CIs containing the national mean rate, and 1 IRF (0.1%) had a 95% CI below the national mean rate.

**Conclusions and Relevance:**

This cohort study found that readmission rates were lower when using the Potentially Preventable 30-Day Post-Discharge Readmission Measure for Inpatient Rehabilitation and further reduced discrimination between facilities compared with the recently discontinued All-Cause Unplanned Readmission Measure for 30 Days Post Discharge From Inpatient Rehabilitation Facilities. This finding may indicate there is a lack of room for improvement in readmission rates. Given the rationale of the Centers for Medicare & Medicaid Services for removing measures that fail to discriminate quality performance, this suggests that the current readmission measure should not be implemented as part of the Inpatient Rehabilitation Quality Reporting Program.

## Key Points

**Question:**

Can Centers for Medicare & Medicaid Services Quality Reporting Program measures detect variation in 30-day hospital readmission rates after postacute inpatient rehabilitation among US inpatient rehabilitation facilities (IRFs)?

**Findings:**

In this cohort study of 454 378 Medicare fee-for-service beneficiaries discharged from 1162 IRFs submitting claims, risk-standardized potentially preventable readmission rates ranged from 4.3% to 7.3%. Application of the Potentially Preventable 30-Day Post-Discharge Readmission Measure for Inpatient Rehabilitation resulted in risk-standardized readmission rates above or below the national mean for less than 1% of 1162 Medicare-eligible IRFs.

**Meaning:**

These findings suggest that the current readmission measure does not distinguish high- and low-performing facilities, and therefore it should not be implemented as part of the IRF quality reporting program.

## Teaser

This cohort study examines the differences between 2 Centers for Medicare & Medicaid Services measures for 30-day rehospitalization after discharge from postacute inpatient rehabilitation.

## Introduction

The Patient Protection and Affordable Care Act^1^ created the Hospital Readmission Reduction Program to reduce the number of readmissions and to increase the success of patient transitions from acute care. While the initial focus was on reducing readmissions after acute care hospital stays, recent attention has been placed on rehospitalizations after postacute care.^2^ A 2019 report from the Medicare Payment Advisory Commission revealed that between 2012 and 2017, risk-adjusted rates of potentially preventable rehospitalizations within 30 days of discharge from inpatient rehabilitation facilities (IRFs) ranged from 4.3% to 4.8%.^3^ Potentially preventable readmissions after an IRF stay are important to all stakeholders, from patients to policy makers, as they expose patients to additional health risks, increase the number of potentially disruptive transitions between settings, and increase Medicare spending.^4^ Thus, addressing post-IRF rehospitalizations has the potential to improve health care quality and reduce costs.

Inpatient rehabilitation facilities serve a critical role in the continuum of care, as they provide intensive rehabilitation and comprehensive medical care with the goal of preparing patients for the highest possible independent living situation at discharge. This goal is met through facilitation of recovery, provision of adaptive equipment and education, and interventions that engage patients in activities required for daily living. Ideally, the patient is discharged to the community (eg, home or supported living) and is able to remain there without a need for readmission to an acute care hospital.^5^

Potentially preventable readmissions (PPRs) to acute care, measured by the Potentially Preventable 30-Day Post-Discharge Readmission Measure for Inpatient Rehabilitation (hereafter, *PPR measure*),^4^ is one of the standardized Centers for Medicare & Medicaid Services (CMS) Quality Reporting Program (QRP) outcome measures specified in the Improving Medicare Post-Acute Care Transformation (IMPACT) Act of 2014,^6^ with public reporting beginning in 2019 for IRFs. This measure was preceded by the All-Cause Unplanned Readmission Measure for 30 Days Post Discharge From Inpatient Rehabilitation Facilities (hereafter, *All-Cause measure*),^7^ which was discontinued at the start of fiscal year 2019. Adoption of the new measure was based in part on evidence suggesting some readmissions could be prevented,^8,9^ especially for certain diagnoses and with appropriate care and discharge planning.^4^ A first step toward reducing PPRs is to examine variations in readmission rates across IRFs, as variation suggests opportunities for improvement may exist. The purpose of this study was to examine facility-level variations in all-cause unplanned and potentially preventable 30-day rehospitalizations after an IRF stay, with the expectation that our findings would provide insight into the ability of these measures to discriminate between well-performing and poorly performing IRFs and could help guide next steps in health care quality initiatives targeting readmissions.

## Methods

This is a retrospective cohort study of Medicare claims data that included 1162 IRFs submitting claims to CMS. We used the Strengthening the Reporting of Observational Studies in Epidemiology (STROBE) reporting guidelines to guide the study analyses and reporting. All analyses were completed after establishing a data use agreement with the Centers for Medicare & Medicaid Services and obtaining approval from the institutional review board of the University of Texas Medical Branch, Galveston, which waived the need for informed consent for use of deidentified data from publicly available files.

### Data Sources

Data were obtained from the Medicare Provider Analysis and Review, Medicare Beneficiary Summary, Medicare IRF-Patient Assessment Instrument, and Medicare Provider of Service. The Medicare Provider Analysis and Review files contain final claims for stays in IRFs, acute care hospitals, skilled nursing facilities, and psychiatric hospitals. We used these files to gather information about patients’ prior hospitalizations, verify their IRF stays, and identify readmissions within the 30-day window after their IRF stay. Medicare Beneficiary Summary files were used to gather Medicare enrollment information. We also used Medicare Beneficiary Summary files to identify patients who died within the 30-day period after their IRF stay. Files were linked using unique, encrypted identifiers.

### Patient Population

**Figure 1.**
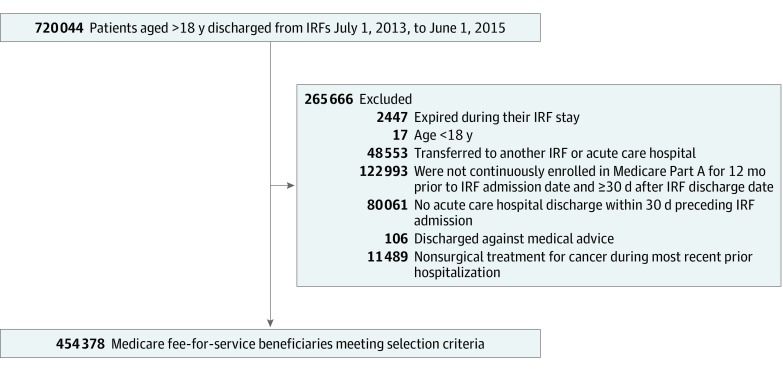
Cohort Selection IRF indicates inpatient rehabilitation facility.

The final cohort included Medicare fee-for-service beneficiaries discharged from an IRF between June 1, 2013, and July 1, 2015 (Figure 1). When separately estimating the all-cause unplanned and PPR rates, we used the exclusion criteria for the All-Cause measure^7^ and the PPR measure,^4^ respectively. The measure specifications exclude patients who died during IRF stay, were younger than 18 years, were transferred to another IRF or acute care hospital, lacked Medicare Part A or had Medicare Advantage during the study period, had no acute care hospital discharge within 30 days preceding IRF admission, were discharged against medical advice, or received nonsurgical treatment for cancer during the prior qualifying hospitalization.

### Outcomes

The main outcomes were the all-cause readmission rate as defined for the All-Cause measure^7^ and PPR rate as defined for the PPR measure.^4^ The All-Cause measure was adopted by CMS in 2015, and the PPR measure was adopted by CMS in 2016. Initially, CMS calculated both readmission measures to monitor all-cause and PPR trends. However, the All-Cause measure was discontinued in fiscal year 2019 to reduce confusion.^10^ The measures differ in the principal diagnoses that count toward a readmission. For the All-Cause measure, the diagnosis must be considered unplanned, while for the PPR measure, the diagnosis must be considered potentially preventable, which is a subset of the All-Cause unplanned diagnoses. A specific list of conditions has been developed for the PPR measure. The included conditions represent inadequate management of chronic conditions, infections, or implanted devices or inadequate injury prevention.^4^ Readmissions associated with these conditions are thought to be avoidable with adequately planned, explained, and implemented postdischarge instructions, including the establishment of appropriate follow-up ambulatory care.^4^

### Statistical Analysis

Data were analyzed March 23, 2018, through June 24, 2019. We calculated risk-standardized rates (RSRs) of all-cause readmissions and PPRs for all IRFs submitting claims to CMS. Risk-standardized rates are used in quality reporting and are based on a standardized risk ratio—the projected number of readmissions for an IRF divided by the expected number of readmissions for the same patients if treated at the typical IRF. Hierarchical logistic regression was used to calculate each IRF’s projected and expected number of readmissions, in which the projected number included the random intercept and the expected number did not. We replicated risk adjustment variables specified for the PPR measure,^4^ namely: patients’ age, sex, original reason for Medicare entitlement (ie, age, end-stage renal disease, or disability), number of acute care stays during the previous year, principal diagnosis or surgical care category from the prior acute stay in the past 30 days, receipt of dialysis in prior acute stay (yes or no), length of prior acute care hospital stay in days for patients or residents whose prior acute care stay was not in a psychiatric hospital (categorical variables are used to account for nonlinearity), an indicator of prior psychiatric hospital stay for patients whose prior acute care hospital stay was in a psychiatric hospital, comorbidities, and inpatient rehabilitation case-mix group. Case-mix groups are based on patients’ impairment category (eg, stroke, lower extremity joint replacement), age, and functional status.^11^ Principal diagnoses and surgical procedures (coded using the *International Classification of Diseases, Ninth Revision* [*ICD-9*]),^12^ were categorized into the Clinical Classifications Software groups (version 22 CMS-HCC risk-adjustment model V2213.79.L2)^13^ developed by the Agency for Healthcare Research and Quality. We categorized comorbidities into the Hierarchical Condition Categories used by CMS. The Hierarchical Condition Categories were assigned based on secondary diagnoses *ICD-9* codes from all hospitalizations during the prior year or the most recent hospitalization according to CMS specifications.^14^ The Clinical Classifications Software groupings and Hierarchical Condition Categories are classification approaches used for principal diagnoses and comorbidities in risk adjustment for the All-Cause and PPR measures.^4^

We used bootstrapping to calculate 95% CI estimates for facility-level RSRs.^4^ These CIs were used to identify IRFs performing significantly better or worse than the mean national rate. To describe facilities performing significantly better or worse than the mean national rate, we accounted for bed count, ownership (ie, government, nonprofit, or for-profit), location (rural or urban), and teaching status (teaching or nonteaching hospital). Facility variables were extracted from CMS Provider of Service files.^15^ Statistical analyses were performed using SAS version 9.4 (SAS institute) and R version 3.4 (R Project for Statistical Computing).

## Results

**Figure 2.**
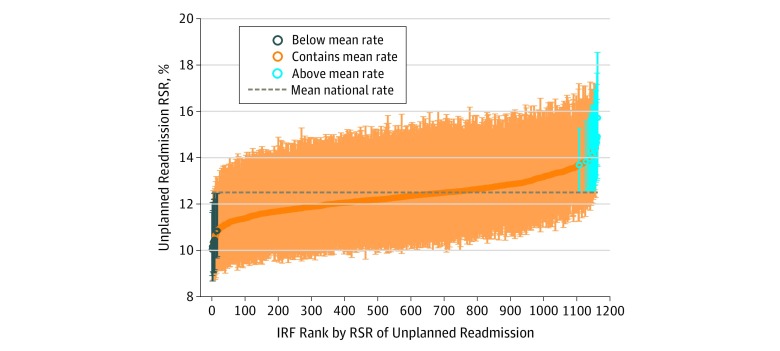
Risk-Standardized Rates (RSRs) of All-Cause Unplanned 30-Day Readmissions After Inpatient Rehabilitation Inpatient rehabilitation facilities (IRFs) are shown in rank order. Circles indicate RSRs; vertical lines, 95% CIs.

**Figure 3.**
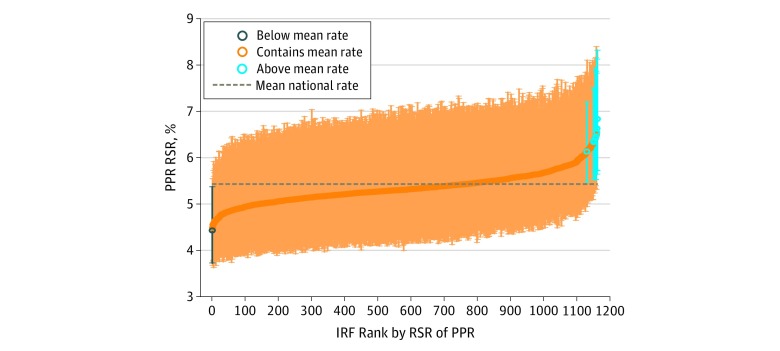
Risk-Standardized Rates (RSRs) of Potentially Preventable 30-Day Readmissions After Inpatient Rehabilitation Inpatient rehabilitation facilities (IRFs) are shown in rank order. PPR indicates potentially preventable readmissions; circles, RSRs; vertical lines, 95% CIs.

Among 454 378 Medicare beneficiaries included, the mean age (SD) was 76.2 (10.6) years; 263 546 (58.0%) were women, 351 235 (81.9%) were white, 42 242 (9.9%) were black, 22 776 (5.3%) were Hispanic, and 12 515 (2.9%) were of other race/ethnicities. The overall all-cause readmission rate after inpatient rehabilitation was 12.3% (95% CI, 12.2%-12.4%). The overall PPR after inpatient rehabilitation rate was 5.3% (95% CI, 5.3%-5.4%). Across the 1162 IRFs, RSRs using the All-Cause measure for readmission rates ranged from 10.1% (95% CI, 8.9%-11.6%) to 15.9% (95% CI, 13.6%-18.6%) (Figure 2), and the RSRs using the PPR measure ranged from 4.3% (95% CI, 3.7%-5.4%) to 7.3% (95% CI, 5.7%-8.3%) (Figure 3). On the All-Cause measure, 16 IRFs (1.4%) were significantly above the mean national rate, 1137 IRFs (97.9%) were within the mean national rate, and 9 IRFS (0.8%) were significantly below the mean national rate. Variation in the PPR measure was even less: only 8 IRFs (0.7%) were significantly above the mean national rate, 1153 IRFs (99.2%) were within the mean national rate, and 1 IRF (0.1%) was significantly below the mean national rate. The limited variation across facilities on both of these measures precluded further analyses by facility characteristics.

## Discussion

Identification of facility characteristics associated with avoidable readmissions after inpatient rehabilitation is a necessary first step in improving health care quality and transitions out of IRFs. The purpose of our study was to examine facility-level variation in all-cause and potentially preventable 30-day rehospitalizations after inpatient rehabilitation using 2 different measures previously adopted by CMS. However, the results of this cohort study demonstrate that the All-Cause measure and the PPR measure each had very limited ability to discriminate between high- and low-performing IRFs related to readmission.

The Patient Protection and Affordable Care Act of 2010^1^ and Improving Medicare Post-Acute Care Transformation Act of 2014^6^ mandated QRPs to incentivize cost savings and improve patient-centered care and outcomes across settings. Unnecessary hospital readmissions have been a particular focus because they are viewed as costly and resource intensive and may expose patients to additional risks or delayed recovery. For example, a 2013 American Hospital Association report^16^ showed that Medicare spending for a representative patient with major joint replacement was $18 128 but increased to $29 803 with readmission to acute care. To reduce readmissions in the 30 days after discharge from an IRF and to hold IRFs accountable for readmissions,^2^ CMS adopted first the All-Cause measure and eventually the PPR measure. The PPR measure has ultimately been favored by CMS, as it focuses on conditions for which a rehospitalization should be avoidable with appropriate discharge planning and follow-up care.^4^

Initially, CMS included both measures in the IRF QRP to track different types of readmissions; however, PPRs are effectively a subset of all-cause readmissions. Indeed, we found the readmission rates using the PPR measure to be less than half of the rate obtained with the All-Cause measure. The overall PPR rate in this study (5.3%) is similar to the 2017 rate reported by the Medicare Payment Advisory Commission (4.7%).^3^ These readmission rates are not directly comparable because of differences in the risk standardization models and updated list of PPR conditions, but they provide some context for our findings.

In this study, use of either the All-Cause measure or PPR measure yielded results which failed to distinguish between high- and low-performing IRFs for 98% to 99% of 1162 facilities included in the analyses. Three key issues may underlie the lack of variation in the measures. First, sociodemographic variables are not included in the algorithm for either measure. A 2014 study by Ottenbacher et al^17^ demonstrated significantly higher post-IRF rehospitalization rates among non-Hispanic black patients compared with non-Hispanic white patients. Although CMS has cautioned that inclusion of sociodemographic variables as a risk-adjustors could unintentionally mask disparities and create different standards of care for IRFs based on a particular IRF’s demographic characteristics, CMS has acknowledged the potential effect of sociodemographic variables and is undertaking a 2-year study^2^ to examine inclusion of sociodemographic risk-factors, the results of which should soon be available.

A second potential limitation of the All-Cause and PPR measures is that functional status is only indirectly controlled for in the risk-standardization models. Case-mix groups are included in the model, and they are largely based on motor functioning, but cognitive functioning is noticeably missing. The study by Ottenbacher et al^17^ demonstrated that across the 6 largest impairment categories receiving IRF care, higher motor and cognitive function ratings were associated with lower readmission rates. Therefore, the Medicare Payment Advisory Commission has suggested functional status measures, such as those used in the Medicare IRF-Patient Assessment Instrument, improve the ability to estimate resource utilization in IRF and other postacute care settings. This is an important area for future research related to post-IRF readmission,^18,19^ and one that CMS reports to be continually monitoring.^2^

A third potential limitation of the All-Cause and PPR measures is the use of the 30-day readmission window, which some investigators have argued is arbitrary.^20,21^ Two studies of Medicare fee-for-service beneficiaries^17,22^ found that a large proportion of patients who were rehospitalized were readmitted within 14 days of their IRF discharge. Earlier readmission may reflect poor coordination of care or poor discharge planning.^21^ Programs implementing early follow-up have reduced readmissions for some patients.^23,24^ Consideration of the timing of readmission may be another important risk-adjustment variable, and would provide incentive to develop appropriate programming, including home visits, integration with primary care, patient and family education, and monitoring technologies.^17^

Potential improvements to the model aside, CMS outlined rationale for removing previously adopted IRF QRP measures in the fiscal year 2019 final rule.^25^ The first factor on the list accurately describes our findings: “Measure performance among IRFs is so high and unvarying that meaningful distinctions in improvements in performance can no longer be made.” ^25^ The lack of variation across IRFs may indicate that the PPR measure has either topped out and that no further meaningful improvements can be made, or that the measure is not sensitive to differences in IRF services. Of the 59 quality measures CMS identified as topped out for the 2019 performance period, the PPR measure was not included.^26^ Our analysis suggests that the IRF PPR measure does not provide meaningful, quality information for consumers or CMS.

### Limitations

This study has some limitations. We used the specifications for the All-Cause and PPR measures to identify the study cohort and calculate RSRs. In addition to not being adjusted for patients’ demographic characteristics, they are not adjusted for Medicaid eligibility. Together, these sociodemographic and economic factors represent social determinants of health that may affect readmission.^27^ Other, unmeasured, and confounding variables may also account for the lack of variation in readmission rates.

## Conclusions

In this cohort study, application of the All-Cause and PPR measures resulted in risk-standardized readmission rates above or below the mean national rate for just 1% to 2% of 1162 Medicare-eligible IRFs. Readmission rates were lower when using the PPR measure and further reduced discrimination between facilities compared with the recently discontinued All-Cause measure. These findings may indicate that there is a lack of room for improvement in readmission rates. Keeping with the rationale of CMS for removing measures that top out or simply fail to discriminate quality performance, we conclude that the current PPR measure should not be implemented as part of the IRF QRP. Future research is needed to develop new rehabilitation-relevant and sensitive measures for tracking inpatient rehabilitation care quality.

## References

[1] MacKinney AC Increases in primary care physician income due to the Patient Protection and Affordable Care Act of 2010—continued tweaking of physician payment. Rural Policy Brief. 2010;111(2010 2):-. 20737732

[2] Department of Health and Human Services, Centers for Medicare and Medicaid Services Medicare Program; Inpatient rehabilitation facility prospective payment system for federal fiscal year 2017 42 CFR Part 412. https://www.govinfo.gov/content/pkg/FR-2016-08-05/pdf/2016-18196.pdf. Accessed June 21, 2019.

[3] Medicare Payment Advisory Commission Report to the Congress: Medicare payment policy. http://www.medpac.gov/docs/default-source/reports/mar19_medpac_entirereport_sec.pdf. Accessed June 21, 2019.

[4] Center for Clinical Standards and Quality Measure specifications for measures adopted in the FY 2017 IRF QRP final rule. https://www.cms.gov/Medicare/Quality-Initiatives-Patient-Assessment-Instruments/IRF-Quality-Reporting/Downloads/Measure-Specifications-for-FY17-IRF-QRP-Final-Rule.pdf. Accessed June 14, 2019.

[5] Middleton A, Graham JE, Prvu Bettger J, Haas A, Ottenbacher KJ Facility and geographic variation in rates of successful community discharge after inpatient rehabilitation among Medicare Fee-for-Service beneficiaries. JAMA Netw Open. 2018;1(7):e184332. doi:10.1001/jamanetworkopen.2018.4332 30646352 PMC6324386

[6] Improving Medicare Post Acute Care Transformation Act of 2014, HR 4994 §113-185 (2014).

[7] National Quality Forum All-cause admissions and readmissions measures: final report. https://www.qualityforum.org/Publications/2015/04/All-Cause_Admissions_and_Readmissions_Measures_-_Final_Report.aspx. Accessed June 21, 2019.

[8] van Walraven C, Jennings A, Forster AJ A meta-analysis of hospital 30-day avoidable readmission rates. J Eval Clin Pract. 2012;18(6):1211-1218. doi:10.1111/j.1365-2753.2011.01773.x 22070191

[9] Vest JR, Gamm LD, Oxford BA, Gonzalez MI, Slawson KM Determinants of preventable readmissions in the United States: a systematic review. Implement Sci. 2010;5:88. doi:10.1186/1748-5908-5-88 21083908 PMC2996340

[10] Department of Health and Human Services, Centers for Medicare and Medicaid Services Medicare Program; Inpatient rehabilitation facility prospective payment system for federal fiscal year 2018 42 CFR part 412. https://www.govinfo.gov/content/pkg/FR-2017-08-03/pdf/2017-16291.pdf. Accessed June 21, 2019.

[11] Department of Health and Human Services Centers for Medicare and Medicaid Services Instructions for Implementing the inpatient rehabilitation facility prospective payment system (IRF PPS). https://www.cms.gov/Regulations-and-Guidance/Guidance/Transmittals/downloads/A0192.pdf. Accessed June 24, 2019.

[12] World Health Organization International Classification of Diseases, Ninth Revision (ICD-9). Geneva, Switzerland: World Health Organization; 1977.

[13] National Bureau of Economic Research Overview of software for the version 22 CMS-HCC risk-adjustment model. http://www.nber.org/risk-adjustment/2014/2014-InitialModel/CMS-HCC_software_V2213.79.L2/HCC%20software%20V2213.79.L2%20description.pdf. Accessed March 23, 2018.

[14] Centers for Medicare & Medicaid Services 2014 Model Software/ICD-9-CM Mappings. https://www.cms.gov/Medicare/Health-Plans/MedicareAdvtgSpecRateStats/Risk-Adjustors-Items/Risk2014.html?DLPage=1&DLEntries=10&DLSort=0&DLSortDir=descending. Accessed June 24, 2019.

[15] Centers for Medicare & Medicaid Services Provider of Services Current Files. https://www.cms.gov/Research-Statistics-Data-and-Systems/Downloadable-Public-Use-Files/Provider-of-Services/. Accessed June 24, 2019.

[16] American Hospital Association Moving towards bundled payment. https://www.aha.org/issue-brief/2013-01-25-moving-towards-bundled-payment. Accessed June 24, 2019.

[17] Ottenbacher KJ, Karmarkar A, Graham JE, Thirty-day hospital readmission following discharge from postacute rehabilitation in fee-for-service Medicare patients. JAMA. 2014;311(6):604-614. doi:10.1001/jama.2014.8 24519300 PMC4085109

[18] Granger CV, Deutsch A, Russell C, Black T, Ottenbacher KJ Modifications of the FIM instrument under the inpatient rehabilitation facility prospective payment system. Am J Phys Med Rehabil. 2007;86(11):883-892. doi:10.1097/PHM.0b013e318152058a 17873825

[19] Deutsch A, Kline T, Kelleher C, Analysis of crosscutting Medicare functional status quality metrics using the Continuity and Assessment Record and Evaluation (CARE) item set: final report. https://aspe.hhs.gov/basic-report/analysis-crosscutting-medicare-functional-status-quality-metrics-using-continuity-and-assessment-record-and-evaluation-care-item-set. Accessed June 24, 2019.

[20] Chen LM, Jha AK, Guterman S, Ridgway AB, Orav EJ, Epstein AM Hospital cost of care, quality of care, and readmission rates: penny wise and pound foolish? Arch Intern Med. 2010;170(4):340-346. doi:10.1001/archinternmed.2009.511 20177036

[21] Joynt KE, Jha AK A path forward on Medicare readmissions. N Engl J Med. 2013;368(13):1175-1177. doi:10.1056/NEJMp1300122 23465069

[22] Leland NE, Gozalo P, Christian TJ, An examination of the first 30 days after patients are discharged to the community from hip fracture postacute care. Med Care. 2015;53(10):879-887. doi:10.1097/MLR.0000000000000419 26340664 PMC4570868

[23] Goodman D, Fisher E, Chang C The Revolving Door: A Report on US Hospital Readmissions. Princeton, NJ: Robert Wood Johnson Foundation; 2013.

[24] Hansen LO, Young RS, Hinami K, Leung A, Williams MV Interventions to reduce 30-day rehospitalization: a systematic review. Ann Intern Med. 2011;155(8):520-528. doi:10.7326/0003-4819-155-8-201110180-00008 22007045

[25] Department of Health and Human Services, Centers for Medicare and Medicaid Services Medicare Program; Inpatient rehabilitation facility prospective payment system for federal fiscal year 2019 42 CFR part 412. https://www.govinfo.gov/content/pkg/FR-2018-08-06/pdf/2018-16517.pdf. Accessed June 21, 2019.

[26] Centers for Medicare and Medicaid Services, Center for Clinical Standards and Quality 2019 Measures under consideration list: program-specific measure needs and priorities. https://www.cms.gov/Medicare/Quality-Initiatives-Patient-Assessment-Instruments/QualityMeasures/Downloads/2019-CMS-Measurement-Priorities-and-Needs.pdf. Accessed June 28, 2019.

[27] Courtney PM, Huddleston JI, Iorio R, Markel DC Socioeconomic risk adjustment models for reimbursement are necessary in primary total joint arthroplasty. J Arthroplasty. 2017;32(1):1-5. doi:10.1016/j.arth.2016.06.050 27506724

